# First person – Bernhard N. Bohnert

**DOI:** 10.1242/dmm.049262

**Published:** 2021-09-15

## Abstract

First Person is a series of interviews with the first authors of a selection of papers published in Disease Models & Mechanisms, helping early-career researchers promote themselves alongside their papers. Bernhard N. Bohnert is first author on ‘ Essential role of DNA-PKcs and plasminogen for the development of doxorubicin-induced glomerular injury in mice’, published in DMM. Bernhard is a physician/postdoc in the lab of Prof. Dr. med. Ferruh Artunc at the University Hospital Tübingen, Tübingen, Germany, investigating oedema formation in nephrotic syndrome.


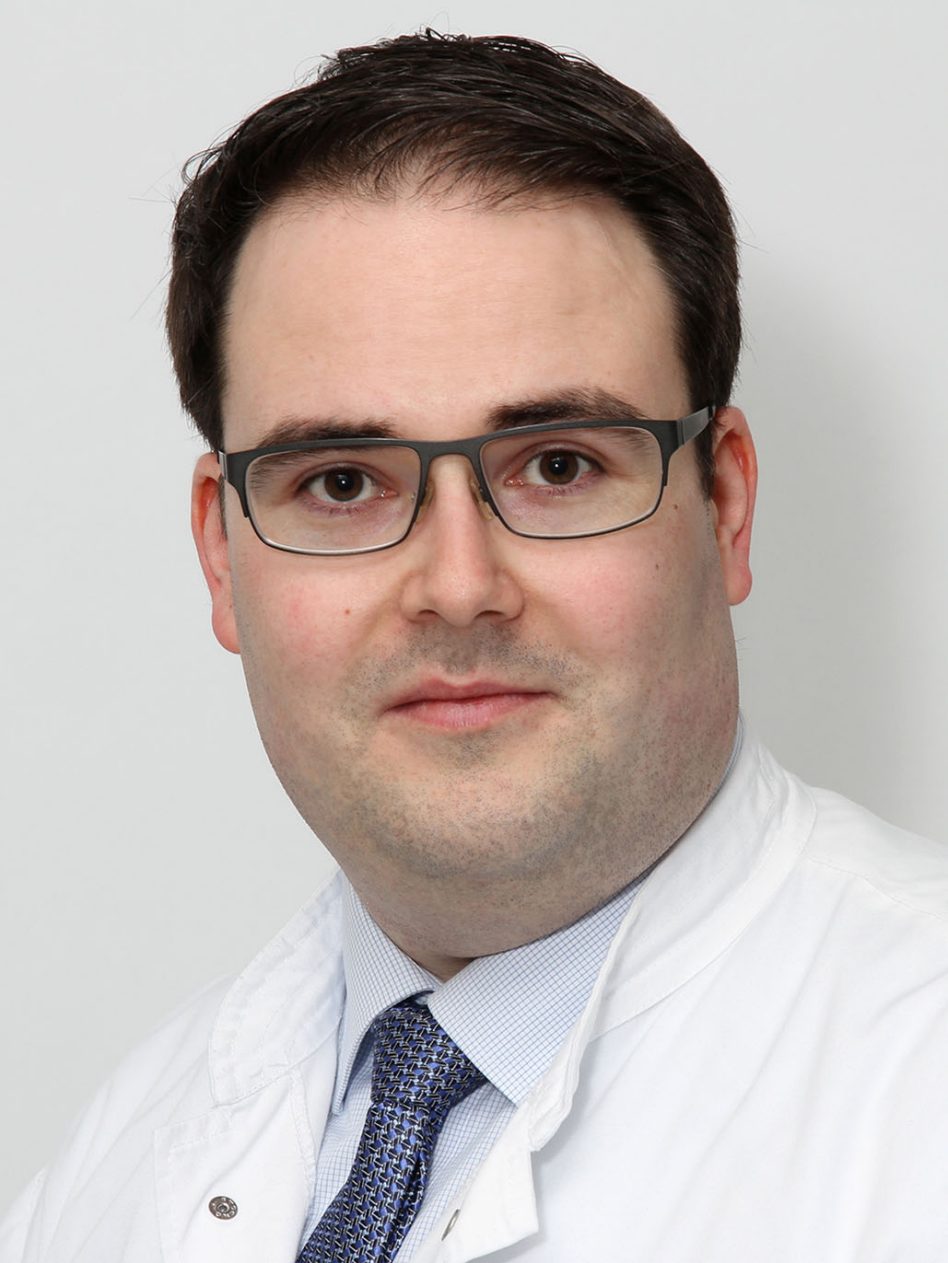


**Bernhard N. Bohnert**

**How would you explain the main findings of your paper to non-scientific family and friends?**

Nephrotic syndrome is a condition that can be caused by various kidney diseases and is characterised by large amounts of proteins leaking to the urine (proteinuria), as a result, low protein levels in the blood (hypoproteinaemia), and massive salt and water retention in the body (oedema). Mouse models can be used to investigate the development of oedema in nephrotic syndrome. One of these models is the model of doxorubicin-induced nephropathy in 129S1/SvImJ mice, a special mouse strain with unique susceptibility to doxorubicin. The present study provides a better understanding of the model, delineating two necessary prerequisites for the successful induction of the model. For the development of nephrotic syndrome after a single administration of the chemotherapeutic agent doxorubicin in mice, a certain genetic variant of a gene is required that plays a relevant role in the repair of the DNA damage induced by doxorubicin. If the variant is present and the DNA damage is not repaired adequately, plasminogen binds to the kidney filter and mediates progression of the insult to nephrotic syndrome.

**What are the potential implications of these results for your field of research?**

The mouse model of doxorubicin-induced nephropathy is a model that, in addition to investigating nephrotic syndrome, also allows investigations of chronic kidney disease and its sequelae, such as proteasuria and oedema formation, development of renal anaemia and secondary hyperparathyroidism. In order to correctly interpret findings obtained in a model and to evaluate their translation to human disease, a precise understanding of the model is required. The work here provides essential prerequisites for the model induction. Furthermore this work builds up on prior work suggesting a direct role for plasminogen as a targetable biomarker and a ‘second hit’ for podocyte injury. It expands further to show the relevance of DNA damage mechanisms. Additionally, the work adds to the ever-growing field showing the likely importance of intra-glomerular communication between podocytes and other cell types.

“The model of doxorubicin-induced nephropathy is a versatile model for chronic kidney diseases and nephrotic syndrome.”

**What are the main advantages and drawbacks of the model system you have used as it relates to the disease you are investigating?**

The model of doxorubicin-induced nephropathy is a versatile model for chronic kidney diseases and nephrotic syndrome. It is one of the few models that generates sufficiently high proteinuria in the mice for sodium retention and oedema to occur, as in patients with nephrotic syndrome. It should be noted that it is a toxic model, which means that the development of glomerular damage in particular cannot be compared with human kidney diseases as there is no such thing as doxorubicin-induced nephropathy in humans. In addition, as already mentioned, the model is limited to a few mouse strains, so that a variation of the background strains is only possible to a limited extent.

**What has surprised you the most while conducting your research?**

To be honest, some of the results obtained are based on an incidental finding. As part of our research on the role of proteasuria in oedema formation in nephrotic syndrome, we aimed to investigate the role of plasminogen in this context. Surprisingly, the plasminogen knockout mice did not become nephrotic, which was, however, not because plasminogen protected against the oedema formation, but, as we found out, because the model could not be induced in the knockout animals as usual. As the animals did not develop proteinuria at all, we took a closer look at our model and collected the interesting findings described in this work. We looked outside the box and were able to learn a lot ourselves.

**Figure:**
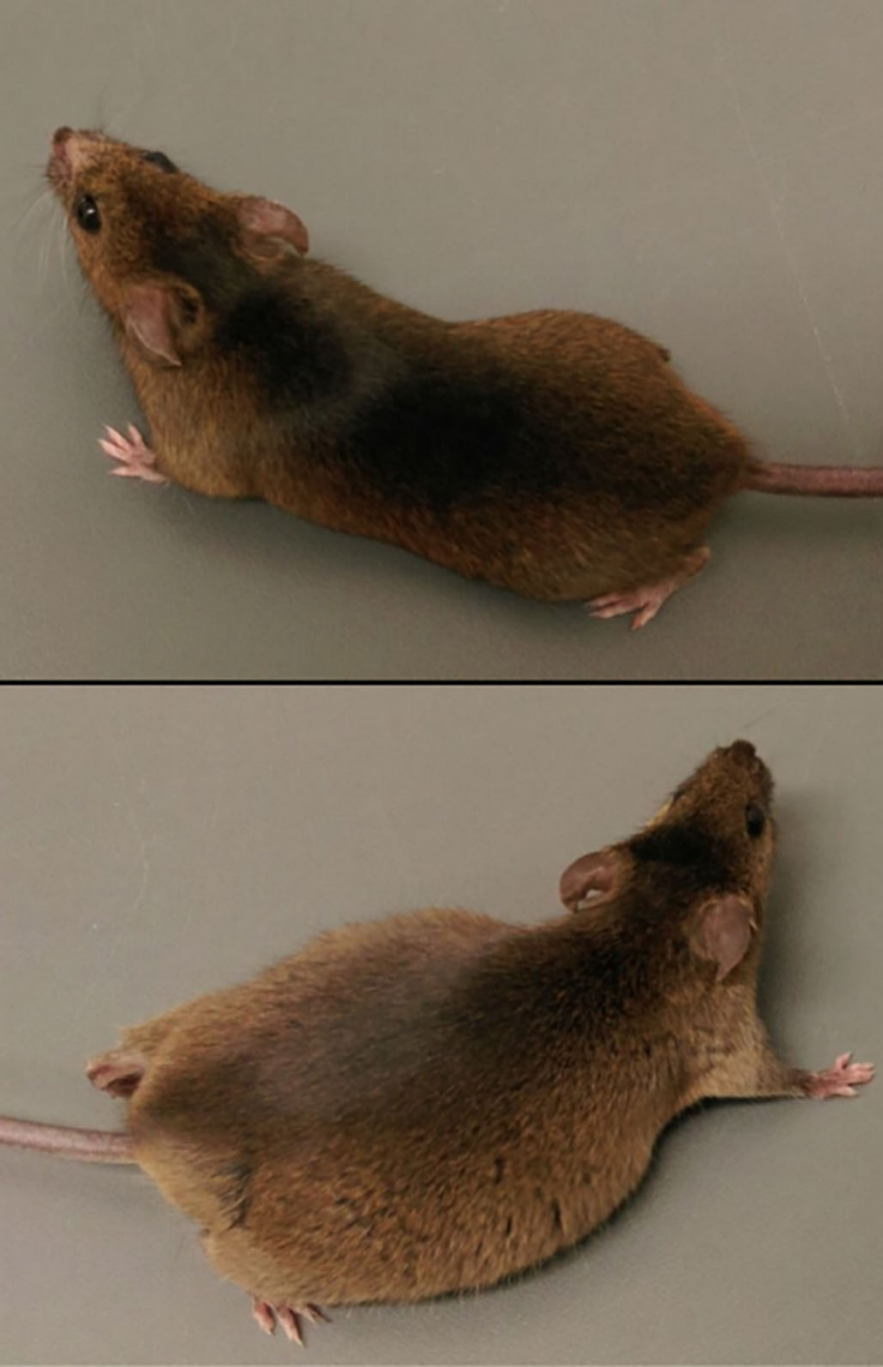
The 129S1/SvImJ mice used in the doxorubicin-induced nephropathy model in healthy (top) and nephrotic (bottom) state. Note the massive oedema in the nephrotic mouse, which can be easily recognised by the prominent flanks that reveal pronounced ascites.

**Describe what you think is the most significant challenge impacting your research at this time and how will this be addressed over the next 10 years?**

We are currently working on a better understanding of oedema formation in the context of nephrotic syndrome. More precisely, we are looking for proteases that are aberrantly filtered by nephrotic kidneys, which we have called proteasuria. Our current challenge is to identify proteases contributing to oedema formation by mediating proteolytic activation of the epithelial sodium channel ENaC expressed in the distal tubule of the kidney. If individual proteases can be identified in the animal model, the aim is to transfer these results to humans and, ideally, to establish new therapeutic strategies for oedema therapy in nephrotic syndrome. For this, however, further studies on animal models and a good understanding of these models are essential.

“[…] a successful career as a young scientist is currently only possible under the care of a mentor who is established in science […]”

**What changes do you think could improve the professional lives of early-career scientists?**

In Germany, there are numerous established programmes for the promotion of young scientists, but there are also some points where I see some potential for improvement. The financing of the research is largely based on third-party funds, which makes it much more difficult for young scientists to carry out independent research without the support of established scientists, since third-party funding can only be obtained by demonstrating successful work that has already been done. This leads to the second point: as young academics in Germany only receive fixed-term employment contracts for years in the university environment, the young people lack the security to plan their scientific future as well as the security in their private lives. In my opinion, a successful career as a young scientist is currently only possible under the care of a mentor who is established in science and under his or her guidance and objectives. From my point of view, facilitating access to financial support for young scientists, improving employment relationships and prohibiting short-term employment contracts for young scientists are points that can promote a scientific career in medicine in Germany.

**What's next for you?**

We will continue finding out which proteases are important in the proteolytic activation of the epithelial sodium channel ENaC in nephrotic syndrome. I am also curious to see what other incidental findings we will stumble upon in the course of this process. Personally, I will concentrate both on my laboratory research and training in nephrology in order to become a clinician scientist committed to cutting-edge research and translation of the findings to patient care.

## References

[DMM049262C1] Bohnert, B. N., Gonzalez-Menendez, I., Dörffel, T., Schneider, J. C., Xiao, M., Janessa, A., Kalo, M. Z., Fehrenbacher, B., Schaller, M., Casadei, N. et al. (2021). Essential role of DNA-PKcs and plasminogen for the development of doxorubicin-induced glomerular injury in mice. *Dis. Model. Mech.* 14, dmm049038. 10.1242/dmm.049038 34423816 PMC8461821

